# Interactions of Some Chemotherapeutic Agents as Epirubicin, Gemcitabine and Paclitaxel in Multicomponent Systems Based on Orange Essential Oil

**DOI:** 10.3390/ph14070619

**Published:** 2021-06-27

**Authors:** Adriana Samide, Bogdan Tutunaru, Renata-Maria Varut, Bogdan Oprea, Simona Iordache

**Affiliations:** 1 Chemistry Department, Faculty of Sciences, University of Craiova, Calea Bucuresti 107i, 200478 Craiova, Romania; samide_adriana@yahoo.com (A.S.); sim_iordache@yahoo.com (S.I.); 2 Faculty of Pharmacy, University of Medicine and Pharmacy, Petru Rareş 2, 200349 Craiova, Romania; rennata_maria@yahoo.com; 3 Faculty of Medicine, University of Medicine and Pharmacy, Petru Rareş 2, 200349 Craiova, Romania; oprea.bogdan@yahoo.com; 4 Faculty of Sciences, Doctoral School of Sciences, University of Craiova, A. I. Cuza 13, 200585 Craiova, Romania

**Keywords:** chemotherapeutics, limonene, interactions, experimental and kinetic studies, quantum chemical calculation, Autodock 4.2.6 system

## Abstract

In order to anticipate the effect induced by a natural product on the chemical activity of medicines simultaneously administered, spontaneous interactions of certain cancer treatment drugs such as, epirubicin (EPR), gemcitabine (GCT), and paclitaxel (PTX) with limonene (LIM)—a natural compound extracted from orange peel and known as an anticancer agent—were investigated. To estimate the stability of the drugs over time, a current density of 50 mA cm^−2^ was applied as an external stimulus between two platinum electrodes immersed in hydrochloric acid solution containing ethyl alcohol/water in the volume ratio of 2/3, in the absence and presence of orange essential oil (limonene concentration of 95%). The concentration variation of chemotherapeutic agents over time was evaluated by UV-Vis spectrophotometry. Kinetic studies have shown a delay in the decomposition reaction of epirubicin and gemcitabine and a paclitaxel activity stimulation. Thus, in the presence of limonene, the epirubicin half-life increased from 46.2 min to 63 min, and from 6.2 min to 8.6 min in gemcitabine case, while for paclitaxel a decrease of half-life from 35.9 min to 25.8 min was determined. Therefore, certain drug-limonene interactions took place, leading to the emergence of molecular micro-assemblies impacting decomposition reaction of chemotherapeutics. To predict drug–limonene interactions, the Autodock 4.2.6 system was employed. Thus, two hydrophobic interactions and five π-alkyl interactions were established between EPR-LIM, the GCT-LIM connection involves four π-alkyl interactions, and the PTX-LIM bridges take place through three hydrophobic interactions and the one π-alkyl. Finally, the decomposition reaction mechanism of drugs was proposed.

## 1. Introduction

The therapeutic potential of plants is known since antiquity, these being used in various ways, e.g., teas, infusions, and local compresses, due to their ameliorating effect on acute or chronic diseases. The pharmaceutical features and the prophylactic/healing qualities were also attributed to certain multicomponent extracts, such as essential oils [1] whose action mechanism on the human body is less repercussive than that of classical pharmaceutical compounds [1,2,3]. The composition of essential oils consists of terpenes and non-terpenes compounds with ability to repress certain tumors [1]. Thus, depending on their suppressive effect on certain diseases, the essential oils are classified into several categories [1] reporting their chemoprophylactic and/or chemotherapeutic activity [2,3,4], the antiviral and antibacterial properties, and cardiovascular effect [1,3,5,6].

The *d*-isomer of limonene with a strong fragrance of oranges is prevailed in oranges-essential oil, reaching a concentration of 95% [7] compared to its *l*-isomer that is mostly found in lemon peel and has a lemon/pine-like odor [7,8]. Limonene is used in the food and pharmaceutical industry to mask the bitter taste of alkaloids, as well as a perfume in the cosmetics or as an adjuvant in cleaning or other personal care products [7,8].

After oral administration, *d*-limonene is rapidly absorbed from the gastrointestinal tract and distributed to the tissues. In the peripheral tissue, it is metabolized by monooxygenases, via cytochrome P450, to oxidized metabolites as perillic acid and dihydroperyl acid and limonene-1,2-diol, cis- and trans-carveol, perillyl alcohol, and limonene-8,9-diol [9,10].

Limonene has been shown to be an effective, non-toxic chemo-preventive and chemotherapeutic agent in chemically induced breast cancer models in rats [9]. The *d*-limonene chemotherapeutic activity can be due to the induction of apoptosis [11,12] and redifferentiation, simultaneously with the increased expression of mannose 6-phosphate/insulin growth factor receptor II and the transformation of B1-growth factor. The *d*-limonene and its plasma metabolites in vivo were shown to be inhibitors of small protein iso-prenylation by G protein, including rasp21 in rats [9]. In addition, to selectively block the iso-prenylation of small G proteins, d-limonene inhibits coenzyme Q synthesis, and causes complete regression of most breast carcinomas in rats without significant toxicity [9,11,12].

This presentation of the limonene therapeutic profile does not imply its widespread use in the cancer treatment. Complementary or unconventional therapies consists of diagnosis and treatment set known as alternative medicine, often controversial and disputed by the allopathic methods that are based on scientific studies and multiple in vivo tests performed over a long time on large groups of patients suffering from a certain disease. Alternative medicine usually considers some case studies with isolated results, assuming that the disease (especially cancer) is caused by certain energy and/or organic imbalances that can be restored by administration of complementary natural remedies. These refer mainly to the healing ability of plant extracts or specific diets prescribed by therapists, often without medical training. In most cases, patients resort to alternative medicine because allopathic treatments have not had the expected results, being overwhelmed by suffering and helplessness, they find a last hope in remedies that do not improve the life quality or anxiety level but can lead to tragic results. However, in some cases, natural remedies can have a placebo effect in pain management, providing physical and mental comfort for a short time to patients who are in the disease terminal stage. Thereby, each person should be aware of both the benefits and risks of alternative therapies, which must be administrated under specialist medical control and should not exclude allopathic treatments.

Chemoprophylaxis acts in the initiation phase of carcinogenesis, and chemotherapy acts in the promotion phase in which the acceleration of tumor cell proliferation occurs [1]. The chemotherapeutic agents (cytostatics) block the growth of cancer cells, influencing the metabolism, so that cell division and reproduction are inhibited. Cytostatics can be classified according to their action mechanism and attack targets. These drugs are generally toxic substances with sometimes dramatic repercussive effects on the body, especially in the first days after administration [13,14,15,16,17,18].

Epirubicin is an anthranilic chemotherapeutic that can be mainly used in various combinations or alone for breast cancer treatment or against ovarian cancer, gastric cancer, lung cancer, and lymphomas [19], acting by intercalating DNA chains, resulting a complex formation inhibiting DNA and RNA synthesis. It also triggers DNA cleavage mechanism causing cancer cell apoptosis. Epirubicin cytotoxic effects are result of its binding to cell membranes and plasma proteins and also generating free radicals causing damage of cells and DNA. It is used in chemotherapeutic treatments to the doxorubicin detriment, as it appears to produce few side effects. The hydroxyl group spatial orientation of 4′ carbon from the carbohydrate group has the opposite chirality, explaining thus the faster elimination and its reduced toxicity compared to doxorubicin.

Gemcitabine is a chemotherapeutic agent classified as nucleoside drug and it is an analogue of cytidine, where two fluorine atoms replace the hydroxyl group on the ribose [20]. It is used to treat of some cancers such as breast, ovarian, non-small-cell lung, pancreatic, bladder, and hematologic disorders like acute leukemia [20]. Additionally, due to its weaker toxic profile compared to other cytostatics, gemcitabine is a good option for pediatric cancer [20]. Gemcitabine acts by blocking a new DNA strand creation which would result in cell death. Common side effects include bone marrow suppression, liver and kidney problems, nausea, fever, transient rash, difficulty breathing, neuropathy, and hair loss, furthermore drug administration during pregnancy it could have teratogenic effects on the fetus. A recently published review [21] presents statistics on adverse reactions to gemcitabine (myocardial ischemia, pericardial disease, arrhythmias, infraction, tachycardia, and heart failure). In most cases, patients were treated with gemcitabine for pancreatic or lung cancer. In the majority of the cases (76–94%) the cardiovascular adverse reactions were severe, and in some cases, the administration was stopped.

Paclitaxel is a chemotherapeutic drug with a taxane structure that acts by interfering with the normal function of microtubules during cell division [22]. Good results were obtained in the treatment of ovarian, breast, lung, cervical, pancreatic cancer, and Kaposi’s sarcoma. Its common side effects include hair loss, bone marrow suppression, numbness, allergic reactions, muscle aches, heart problems, increased risk of lung infection and inflammation, and administration during pregnancy can cause congenital malformation of the fetus.

The combination therapies are useful for treatment of end-stage breast cancer patients whose can no longer be intensively administrated the chemotherapy and radiation therapy, and when a motivation to combine herbal constituents with synthetic medication is well-founded [23,24]. The combination of plant extracts with synthetic therapy improves bioavailability, facilitates transport, reduces the effective dose, changes the biological activity on therapy-resistant bacteria, etc. [24]. Therefore, reported data [23,24] showed a promising therapeutic potential for the breast cancer treatment using a combination of paclitaxel with curcumin, baicalein and resveratrol by into synergic growth inhibition induces apoptosis in MCF-7 levels in breast tumors of mice [25,26].

Based on in vitro and in vivo experimental data, it was hypothesized that a combination therapy between *d*-limonene and docetaxel can induce a synergistic effect in inhibiting apoptosis signaling pathways, during cell growth, in prostate cancer [27]. The study highlighted in vitro antitumor potential of *d*-limonene in combination with docetaxel, using DU-145 cells from human prostate cancer [27].

Essential oil of leaf of Tithonia Diversifolia (Hemsl) A. Gray, which also contain limonene, have been studied for its antioxidant and antimicrobial activity [28]. The authors obtained an antioxidant activity of 54.6% for a concentration of 5 mg mL^−1^ and a value of 4.30 for IC50 [28]. Curcumae Rhizome essential oil can inhibit cell proliferation, induce cell apoptosis and stop the cell cycle in human cervical carcinoma cells by reducing PTEN, AKT, and STAT3 phosphorylation and down-regulating signaling of NF-κB [29]. β-elemene (components of the Curcumae Rhizome essential oil) may increase the cytotoxicity and sensitivity of cisplatin in various types of carcinomas. The combination of the drug medication with herbs has a long history. Some of the drug-plant interactions can increase the therapeutic effect or reduce the side effects of the drugs [29,30,31,32,33]. Recent studies have shown that the concomitant use of *S. baicalensis* extract and cisplatin for the treatment of ovarian cancer has increased the effectiveness and reduced the side effects of the chemotherapeutic [30]. Not only herbs, but also food and dietary supplements can interact with anticancer drugs via cytochrome p450 isoforms 3A4, 2D6, 1A2, and 2C8 [31]. This study systematizes data from the literature and shows that approximately half of anticancer drugs are substrates for 3A4 and have pharmacokinetic interactions [31]. Authors study the interactions of over 50 chemotherapeutics and over 250 herbs, food and dietary supplements and proves that some of them are inhibitor, inducer or have no interaction with CYP3A4. A systematic analysis of inhalation aromatherapy and aromatherapy massage improves various physical and psychological complications of cancer patients [32].

The present study aims to analyze the limonene impact on the activity of some cytostatics, such as epirubicin (EPR), gemcitabine (GCT), and paclitaxel (PTX), in order to determine the specific interactions between limonene and chemotherapeutic agents, in storage environments before dilution and administration in infusion solutions. Thus, the stability of the drugs is guaranteed before the application of external stimulus, being avoided additional interactions with the basic environment and/or the appearance of secondary influencers during electrochemical measurements. Therefore, the interactions of limonene (LIM) with each chemotherapeutic agent (CYT), respectively EPR, GCT, and PTX were studied in the mixed hydrochloric acid/ethanol solution, in the absence (SE) and in the presence of orange essential oil (ESO) using electrochemical measurements assisted by UV-Vis spectrophotometry. Chosen study environment based on 5 × 10^−2^ mol L^−1^ hydrochloric acid solution containing ethylic alcohol/water, in a ratio of 2/3 (volumes) ensured the optimal solubility of orange essential oil, respectively limonene and also allowed the evaluation of drug–ethanol interactions.

The experimental measurements were supplemented by theoretical studies based on which the molecular descriptors specific to each compound were determined, further predicting the type of interactions that can be established between limonene and the studied chemotherapeutic agents.

## 2. Results and Discussion

### 2.1. Experimental Results

As noted in Section 3, the drug–orange essential oil interactions, over time, were studied in a supporting electrolyte (SE) consisting of 5 × 10^−2^ mol L^−1^ HCl in ethyl alcohol/water solution, in volume ratio of 2/3, using constant current density electrolysis assisted by the UV-Vis spectrophotometry. Comparatively, three distinct environments were analyzed and for which the most appropriate kinetic models were applied. The analyzed environments will be further named, as follows: (i) CYT_HCl, where CYT represents, in turn, epirubicin (EPR), gemcitabine (GCT), and paclitaxel (PTX); (ii) CYT_SE, respectively, EPR_SE, GCT_SE, and PTX_SE; (iii) CYT_ESO, respectively, EPR_ESO; GCT_ESO; and PTX_ESO, where ESO consists of SE containing orange essential oil.

#### 2.1.1. Spectrophotometric Study

The UV-vis spectra collected for each drug, in 5 × 10^−2^ mol L^−1^ HCl solution, in SE and in ESO, are systematized in Table 1.

According to literature reported data, the orange essential oil spectrum obtained in SE displays, for limonene (LIM), a peak at 274 [34,35] followed by a smaller one at 252 nm (the first row from Table 1). The splitting of absorption maximum can be caused especially by the appearance of hydrophobic interactions and/or due to the limonene susceptibility to oxidation, during storage, at one or both double bonds leading to the formation of some compounds such as oxides, peroxides, or alcohols [36,37].

As mentioned in the experimental part, the orange essential oil contains as majority component limonene in concentration of 95.86% and up to 100% other compounds in percentage range from 0.12% to 2% (the last, only *β*-myrcene). For the accuracy of the spectrophotometric response, in all determinations the orange essential oil was diluted by 10,000 times. The additional compounds are in very low concentration (traces), in all studied environments, below the detection limit.

However, the interactions of chemotherapeutic agents with these compounds are not excluded, but those with limonene will be discussed as a matter of priority, being the component that significantly influences the analyzed system.

For epirubicin in HCl solution (EPR_HCl), a peak centered at 500 nm on UV-Vis spectrum was recorded (the second row of Table 1), being in full agreement with other studies [38,39]. In case of alcoholic HCl solution, EPR_SE system, some changes of the EPR spectral characteristics are highlighted, involving the occurrence of an irregular peak shifted to absorbance value higher than the one observed in the aqueous HCl solution, but without wavelength alteration. Thus, strongly interactions between epirubicin and ethyl alcohol take place that affect both the accuracy of absorption maximum as well as its surface. 

The essential oil addition in SE (EPR_ESO) slightly modified the epirubicin spectrum in the sense that the absorbance value insignificantly increases but the peak fluctuations are more intense, while the LIM corresponding peak, at 274 nm, is no longer detected. These changes can be caused mainly by certain EPR-LIM interactions overlapped on those between EPR and ethyl alcohol, the latter prevailing, including to the detriment of secondary interactions with other compounds from essential oil composition.

From the Figure inserted in Table 1, the third row, it can be observed that in both aqueous and alcoholic HCl solutions gemcitabine (GCT) absorption maximum is located around 278 nm, what it is in agreement with literature data [40,41]. The spectral characteristics of the drug are similar in the two systems, GCT_HCl and GCT_SE, suggesting that, there are no obvious interactions between gemcitabine and ethyl alcohol. 

Contrary, in ESO, the spectrum displays a peak whose maximum is shifted to a higher absorbance value than the previous ones and which is formed by the additive overlapping of the two maxima corresponding to limonene (274 nm) and gemcitabine (278 nm). The randomly divided peak as well as the completely disturbed spectrum can indicate the appearance of certain interactions between the two compounds, leading to formation of some GCT-LIM intermolecular bonds simultaneously with the ones due to particular essential oil compounds.

In both HCl solution and SE, paclitaxel (PTX) shows a single absorption maximum at 230 nm (the fourth row of Table 1), as reported in the literature [42]. The minor differences between the two spectra suggest that the interactions between PTX and ethyl alcohol are less perceivable, which is expected because paclitaxel solution for injection contains ethanol, as an excipient.

In the presence of ESO, PTX peak from 230 nm is altered by numerous noises, being preceded by the limonene spectrum under a shoulder shape, as proof that a part of it was involved in the appearance of some PTX-LIM interactions. These can superimpose with cumulative interferences of other oil compounds absorbing at wavelengths less than 250 nm, as well as with the ones of macrogolglycerol ricinoleate-excipient from PTX solution take place.

#### 2.1.2. Electrochemical Study

**Figure 1:**
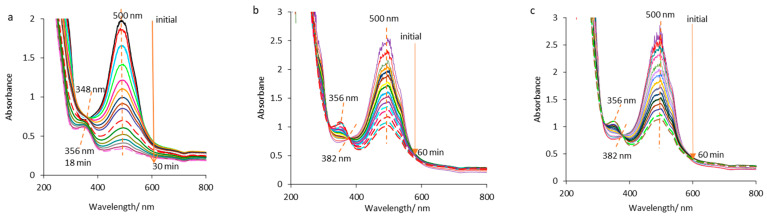
Epirubicin UV-Vis spectra recorded during electrolysis at a constant current density of 50 mA cm^−2^: (**a**) in aqueous HCl solution (EPR_HCl); (**b**) in supporting electrolyte, in the absence of essential oil (EPR_SE); (**c**) in supporting electrolyte, in the presence of essential oil (EPR_ESO).

**Figure 2:**
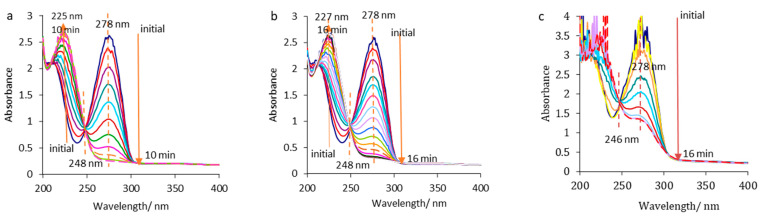
Gemcitabine UV-Vis spectra recorded during electrolysis at a constant current density of 50 mA cm^−2^: (**a**) in aqueous HCl solution (GCT_HCl); (**b**) in supporting electrolyte, in the absence of essential oil (GCT_SE); (**c**) in supporting electrolyte, in the presence of essential oil (GCT_ESO).

**Figure 3:**
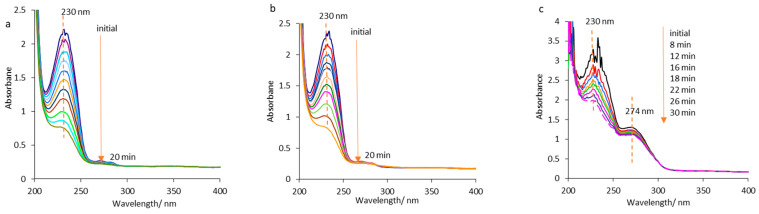
Paclitaxel UV-Vis spectra recorded during electrolysis at a constant current density of 50 mA cm^−2^: (**a**) in aqueous HCl solution (PTX_HCl); (**b**) in supporting electrolyte, in the absence of essential oil (PTX_SE); (**c**) in supporting electrolyte, in the presence of essential oil (PTX_ESO).

To evaluate the effect of orange oil on the electrochemical behavior of chemotherapeutic agents to determine the influence of orange essential oil on their decomposition reaction, electrolysis at constant current density was applied assisted by UV-Vis spectrophotometry. UV-vis spectra of drugs in aqueous HCl solution (CYT_HCl), in supporting electrolyte consisting of alcoholic HCl solution (SE), in the absence (CYT_SE) and in the presence of orange oil (CYT_ESO) are shown in Figure 1, Figure 2 and Figure 3.

Figure 1a shows the epirubicin spectra in aqueous HCl solution (λ_max_ = 500 nm) recorded during electrolysis, every 2 min, indicating that it degrades almost completely in 30 min. The absorbance declined from 2 value to 0.34, and consequently the concentration decreased from 2.57 × 10^−4^ mol L^−1^ to 4.37 × 10^−5^ mol L^−1^ (both, approximately of 5.9 times) very close to the EPR detection limit.

The isosbestic point at 348 nm persists time of 18 min, after which a new compound, at a wavelength of 356 nm is highlighted on EPR_HCl UV-Vis spectra, its concentration increasing gradually until the final moment.

Epirubicin decomposition in SE (Figure 1b) is slower than that in aqueous HCl solution, the absorbance decreasing about of 2.8 times, after 60 min, thus confirmed the existence of certain interactions between ethyl alcohol and epirubicin. The characteristics of spectra (registered every 4 min) are changed (Figure 1b) illustrating that the isosbestic point is shifted at 382 nm and persists throughout the electrolysis, while the compound from 356 nm is gradually formed as the decomposition reaction of EPR takes place.

In the presence of essential oil (Figure 1c), similar spectra to the previous ones (Figure 1b) were recorded during electrolysis, and apparently, EPR electrodecomposition reaction inhibition takes place, the absorbance dropping by 2.5 times, after 60 min.

Figure 2 illustrates the gemcitabine UV-Vis spectra recorded minute by minute during electrolysis in aqueous HCl solution (Figure 2a) and in supporting electrolyte (Figure 2b). Figure 2a (GCT_HCl) and Figure 2b (GCT_SE) show that, in the absence of essential oil, gemcitabine decomposes in a short time of 10 min in aqueous HCl solution and in 16 min in alcoholic HCl solution (SE) without the wavelength alteration of the absorption maxima. After 10 min, in the case of the GCT_HCl system the absorbance drastically declines and implicitly the concentration decreases under detection limit compared to the GCT_SE system, when the absorbance lowers after 16 min and GCT is no longer detectable. The isosbestic point at 248 nm appeared, usually indicating that, only two species varying in concentration contribute to absorption around this wavelength but implying a certain stoichiometric ratio such that the absorbance to be invariable.

From the gemcitabine spectra recorded every two minutes in ESO, the intensity of the GCT apparent peak detected at 278 nm decreases over time (Figure 2c) simultaneously with the limonene concentration drop. Significant spectral changes took place, such as the disappearance of spectral fluctuations after 6 min and GCT detection after 16 min as a descending shoulder, involving the concentration decrease followed by the isosbestic point formation at 246 nm. The spectra recorded at 14 and 16 min overlapped indicating the system stability, probably due to the formation of molecular microaggregates framing the gemcitabine molecules.

Thus, orange essential oil does not influence the GCT decomposition mechanism but has an electrocatalytic activity on the decomposition reaction of new compounds detected at 225–227 nm. In this case, it can be assumed that some GCT-LIM bonds were formed and a mutual influence on their decomposition rate happened. A synergistic action of the two components inhibiting their individual activity can be suggested.

The behavior of paclitaxel in hydrochloric acid solution, in the absence (PTX_HCl) and in the presence of ethyl alcohol (PTX_SE) can be observed in the spectral images designed in Figure 3a,b. In both cases, the paclitaxel shows a peak at 230 nm, classically decomposing, without changing the characteristics and wavelength of the maximum absorption.

The PTX decomposition evolved 20 min in hydrochloric acid solution, with a visible decrease in absorbance at every two minutes of sampling during electrolysis. Finally, PTX absorption maximum reaches a value of 0.82 corresponding to a concentration of 4.3 × 10^−6^ mol L^−1^, this being about of 2.7 times smaller than the initial one (1.17 × 10^−5^ mol L^−1^). The ethyl alcohol presence leads to the decomposition reaction delay, prolonging the reaction time and an absorbance depletion from 2.34 to 0.79. In the presence of orange essential oil (PTX_ESO), seemingly strong interactions/interferences between paclitaxel and limonene take place. As a result, the peak of drug is unnoticed, and the numerous splits of the absorbance maxima lead to drastically perturbation of PTX spectra, declining during electrolysis simultaneous with limonene concentration (Figure 3c). In this case, the orange essential oil accelerates the paclitaxel breakdown, thus affecting its spectrophotometric response.

As stated above, paclitaxel Actavis contains castor oil (macrogolglycerol ricinoleate) and ethyl alcohol. Thus, an excess of ethanol can have unwanted side effects on its activity and macrogolglycerol ricinoleate can interact/interfere with limonene or other essential oil compounds.

In the case of all chemotherapeutic agents, the occurrence of decomposition compounds that can absorb at the same wavelength is unlikely. The additional absorption would increase the absorbance values due to the interference of the several spectra, which would be also visible at the end of the process, when a large amount of decomposition products is accumulated. As shown above, certain decomposition products are displayed at shorter wavelengths than the respective drug one.

Consequently, orange essential oil inhibited the decomposition reaction of epirubicin and gemcitabine and a retarding effect on each to other on decomposition reaction probably happened, due to some interactions that favored the instantly formation of drug-limonene bridges. The behavior of the two cytostatics during electrolysis suggested the formation of molecular micro-assemblages based on stronger bonds between GCT-LIM than those EPR-LIM. Orange oil accelerated the paclitaxel chemical degradation from the first moment of drug-oil contact. However, it is difficult to estimate the spontaneous formation of a PTX-LIM molecular complex, which could lead to a delay in its decomposition reaction.

Moreover, the essential oil effects on the electrochemical stability of the three drugs synergistically overlap on ethanol ones, the latter stronger affecting epirubicin than gemcitabine and paclitaxel.

#### 2.1.3. Kinetic Approach

To evaluate quantitatively the electrochemical decomposition reaction of cytostatics in 5 × 10^−2^ mol L^−1^ HCl blank solution (CYT_HCl), in supporting electrolyte without (CYT_SE) and with orange essential oil (CYT_ESO), the kinetic models of zero-order reactions and first-order reactions were applied. For a comparative approach, the half-life was determined, according to the kinetic model fitting the best the experimental data.

The rate laws corresponding to kinetics of zero and first-order reactions are represented by the expressions (1) and (2), respectively [43,44,45].

A = A_o_ − kt (1)

ln(A_o_/A) = kt (2)

where, A_o_ and A represent the absorbance values at initial time and at a certain reaction time, respectively; k is the rate constant; and t represents the reaction time.

As it can be seen from Equation (1), by the graphical representation A = f(t), a straight line with a negative slope equal to −k, and the intersection with the ordinate representing absorbance initial value (A_o_) is obtained. Applying Equation (2), the plot ln(A_o_/A) = f(t) designs a straight line that passes through origin, its slope being equal with k.

In the case of zero-order kinetics the absorbance variation over time is also represented by the Equation (1), while the one corresponding to the first-order kinetics is deduced from the Equation (2) obtaining the following expression (3):

A = A_o_e^−kt^ (3)

The half-life (t_1/2_) represents the reaction moment at which the reagent initial concentration decreased twice, and consequently the absorbance value decreased by half. The half-life expressions deduced from the respective laws are presented below, relation (4) for the kinetics of zero-order reactions and (5), respectively, for that of the first-order reactions.

t_1/2_ = A_o_/2k (4)

t_1/2_= (ln2)/k (5)

**Figure 4:**
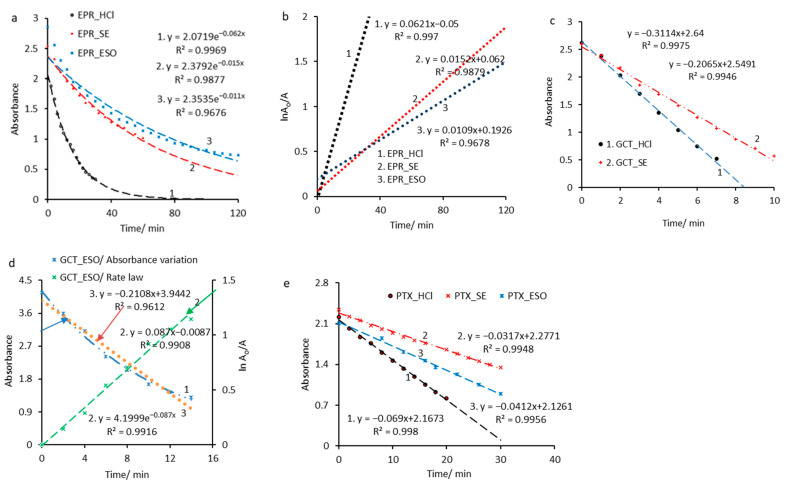
Kinetic model approached for the electrodegradation reaction of chemotherapeutics: (**a**,**b**) kinetics of first-order reactions applied on the epirubicin decomposition in all studied media; (**c**) kinetics of zero-order reactions applied on gemcitabine decomposition in HCl blank solution and in SE; (**d**) kinetics of first-order reactions applied on gemcitabine decomposition in the essential oil presence (ESO); (**e**) kinetics of zero-order reactions applied on the paclitaxel decomposition in all the three media.

The kinetic models that fitted most appropriately the experimental data, with a coefficient R^2^ very close to unity are shown in Figure 4. Thus, from Figure 4a it is observed that, in the case of epirubicin decomposition reaction, in all studied media, EPR_HCl, EPR_SE, and EPR_ESO, the absorbance varies exponentially over time, respecting the kinetics of the first-order reactions. It should be noted that, in the case of the EPR_ESO system, the coefficient R^2^ reaches the value of 0.967. This deviation requires a designation closer to reality for EPR decomposition in ESO, such as pseudo-first-order reaction kinetics.

The rate constants were computed from the inserted equations in Figure 4a, representing “e” exponent number, reaching approximately similar values with those determined from the rate law (Figure 4b), by deriving the equations corresponding to each media. Moreover, for the initial absorbances very close values to the experimental ones were obtained (Equations from Figure 4a—the digit from “e” front).

Additionally, a suitable kinetic pattern can be applied to GCT decomposition reactions (Figure 4c,d). The absorbance declines over time, in all the three media, changing its linear decrease tendency for GCT decomposition in HCl blank solution and in SE (Figure 4c) with the exponential trend for GCT decomposition in ESO. Thus, a zero-order kinetics was designed for the specific electrodegradation reactions in HCl blank solution and in HCl solution containing ethanol (Figure 4c). In contrast, the presence of essential oil leads to a change in the kinetics from zero to the first-order reaction (Figure 4d) indicating a deeper retardation of the GCT decomposition reaction. However, some additional explanations are needed involving more in-depth discussions. It should be noted that, by applying a kinetic model afferent to the zero-order reactions (Figure 4d—line 3), the coefficient R^2^ is lower than in the previous case, and the initial absorbance value is further away from the experimental one (Figure 4d—Equation (3)).

As shown in Figure 4e, the electrodegradation reaction of paclitaxel classically follows a zero-order kinetics, in all three environments.

The experimental/computed absorbance values and the mean square deviation (R^2^) are comparatively presented in Table 2.

According to the data from Table 2, kinetic model of first-order reactions is more substantial for EPR degradation in HCl blank solution and in SE compared to EPR decomposition in ESO. More or less hypothetical, this deviation is due to instantaneous interactions that occur from the EPR-oil first contact (Section 2.1.1, Table 1, second row), delaying the decomposition reaction for a time of 12 min (comparison between Figure 1b,c), thus leading to the kinetics model disturbance from first to pseudo-first-order.

The reaction rate (-dA/dt) of gemcitabine decomposition in HCl blank solution (GCT_HCl) and in supporting electrolyte (GCT_SE), is independent of the drug concentration, at a certain time, being dependent only on the rate constant.

GCT decomposition does not instantly occur on the first its contact with orange essential oil, the two species assembling through some bonds/bridges into a stable molecular microaggregate, as shown in Section 2.1.2, Figure 2c. This GCT-LIM connection can cause a slower drug degradation with a reaction rate (-dA/dt) dependent on its concentration, at a certain decomposition reaction time. As shown in Figure 2c (Section 2.1.2), the irregular and persistent fluctuations of absorption maximum, time of 6 min, can suggest that GCT-LIM bonds or bridges with other particular essential oil compounds were formed, impacting on drug electrochemical stability. Thus, the approach of a first-order kinetics for the GCT decomposition reaction, in the presence of essential oil, would better reflect the GCT-LIM assembling, than the zero-order pattern.

As shown in Section 2.1.1 and Section 2.1.2, ethyl alcohol interacts more strongly with epirubicin than with gemcitabine, causing a significant retard of EPR decomposition reaction compared to GCT the one. The orange essential oil presence in the supporting electrolyte leads both to the rate constant decrease and reaction order change, from first to pseudo-first order, in case of EPR and from zero-order to first-order for GCT, indicating that the electrochemical degradation of each drug was delayed.

Contrary, paclitaxel decomposition reaction is slightly accelerated by orange essential oil presence in SE and less controllable than the previous ones.

Additionally, the presence of ethanol in the HCl solution leads to an increase in half-life for all drugs. The addition of essential oil in SE determines an increase in the half-life of GCT and EPR, but a less value for PTX was obtained compared to that calculated in its absence. The increase of half-life shows that it is necessary an additional effort for the cleavage of CYT-LIM bridges or other bonds created with certain specific oil compounds for the release of the chemical species in the reaction environment, thus their decomposition subsequently taking place. Decreasing of half-life causes apparently uncontrolled increase of paclitaxel reactivity.

The comments are supported by the computed values for rate constants and half-lives from the kinetic models approached for the electrodegradation reaction of drugs in HCl blank solution, in SE and ESO, as shown in Table 3.

Analyzing the data from Table 3, it is observed that the electrodegradation reaction rate constants of drugs, in supporting electrolyte (SE), range as follows: kGCT>kPTX>kEPR, and the half-life increases in the opposite direction: t_1/2_(EPR)>t_1/2_(PTX)>t_1/2_(GCT), which is in line with those discussed in the previous Section 2.1.2.

Orange essential oil causes a retarding effect on both the decomposition reactions of gemcitabine and epirubicin but potentiates the paclitaxel decomposition.

Thus, GCT passes through from relative instability (k = 0.2065 uA min^−1^) in SE, to a better stability in ESO (k = 0.081 min^−1^), due to especially the formation of GCT-LIM molecular microaggregates assembled by relatively hardly destructible bridges, even when applying an aggressive external stimulus as the electric current. In the case of epirubicin, the ethanol action is predominant, the essential oil effect being attenuated, thus causing a limited delay in decomposition reaction, as shown the rate constant variation, from 0.015 min^−1^ to 0.011 min^−1^, indicating that EPR-LIM predominant connections are more labile compared to those of GCT-LIM. PTX presents a high susceptibility in essential oil presence, which annihilates the spectral characteristics, leading to an increase of constant rate from 0.0317 uA min^−1^ to 0.0412 uA min^−1^ and a depletion of half-life from 35.9 min to 25.8 min.

Taking into account the above, it can be concluded that the presence of essential oil in supporting electrolyte leads to: (i) EPR decomposition reaction relative inhibition; (ii) a delayed effect on GCT decomposition; (iii) instantaneous action on PTX due to the synergistic effect of limonene and other oil compounds that absorb at wavelengths less than 250 nm, e.g., *β*-myrcene, accelerating its decomposition reaction.

Metabolic decomposition (breakdown) of drugs by living organisms is biocatalyzed by enzymes and involves many pathways through which the chemical structure of drugs is altered. These are foreign compounds of basic metabolism biochemistry, and the biotransformation involves the knowledge of the mode, rate, and degree of absorption, metabolic breakdown and elimination rate, the amount stored in organs, cumulative actions, and the sensitivity of animal species subjected to the experiment. It is imperative to study the nature of macroscopic and microscopic changes as well as the doses administered at which certain effects occur. Certainly, pharmacokinetics is different from chemical kinetics as a way of approaching, but the results obtained in simulated laboratory environments can be relevant. Thus, chemical kinetics can constitute a starting point regarding decomposition rates and half-lives of drugs, especially since the multitude of internal and external factors that act on living organisms can alter the expected biological response, and the hazard is sometimes relentless.

### 2.2. Theoretical Study

**Figure 5:**
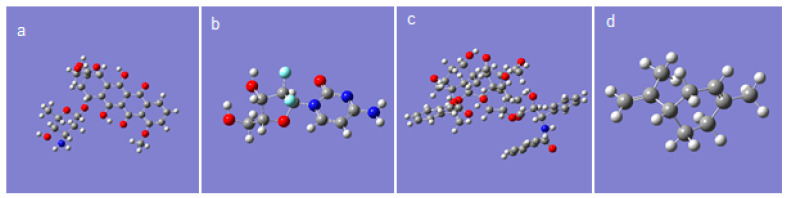
Molecular structures of studied chemical compounds: (**a**) epirubicin; (**b**) gemcitabine; (**c**) paclitaxel; (**d**) limonene.

In order to complete the information obtained from the experiments, a theoretical study was performed on the interactions between the studied chemotherapeutics and limonene, this being the majority component from orange essential oil. The molecular structures of limonene and the drugs shown in Figure 5 were obtained, and various structural descriptors that can provide information on the chemical activity and a possible biological response of the studied compounds were computed.

#### 2.2.1. Analysis of HOMO-LUMO Frontier Orbitals

The analysis of frontier orbitals, HOMO (the highest occupied molecular orbital) and LUMO (the lowest unoccupied molecular orbital) leads to information about the interaction types between molecules based on donor-acceptor electron transfer processes, involving the OMO layer or UMO one, respectively. The energy value of HOMO (*E*_HOMO_ descriptor) allows an appreciation on the electron-donating capacity by a molecule and its oxidation tendency, respectively. Instead, the *E*_LUMO_ value indicates the electron-accepting affinity by a molecule and its reduction tendency, respectively. A high *E*_HOMO_ value denotes the molecule susceptibility to electrophilic attack, while that of *E*_LUMO_ high value signals the molecule propensity to nucleophilic attack.

**Figure 6:**
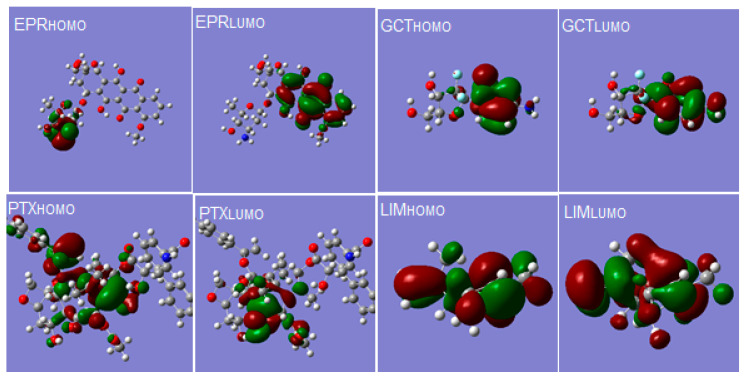
Frontier orbitals, HOMO and LUMO, of the studied compounds.

The energy gap between the HOMO and LUMO levels (Δ*E* = *E*_LUMO_ − *E*_HOMO_) is also a chemically important molecular descriptor showing the molecule stability, its low values being specific to soft molecules, with high chemical reactivity [46]. Frontier orbitals, HOMO and LUMO of the studied compounds are presented in Figure 6 and molecular descriptors are shown in Table 4.

Based on energy gap values, it can assume that molecular stability varies as follows: GCT > PTX > EPR, thus explaining the lower susceptibility of gemcitabine and paclitaxel in the presence of ethanol compared to that of epirubicin.

However, the solvent can destabilize the frontier molecular orbitals, HOMO and LUMO, leading to the modification of ΔE values compared to those obtained in gas or vacuum [47]. Moreover, the application of an external impulse, such as an electric current, can induce certain deviations from chemical stability order identified in gas or vacuum, thus facilitating a faster decomposition of gemcitabine than that of paclitaxel and epirubicin, respectively. Knowing that injectable epirubicin and gemcitabine are stored in aqueous solutions containing natrium chloride and hydrochloric acid, their stability in the presence of Cl^−^ anions is undeniable, but it is significantly influenced by the electric current applied between two platinum electrodes.

Commonly, the predetermined conditions in the human body can change the pharmacokinetics and dynamics of chemotherapeutic agents and consequently their biological response, especially if these are administered in drug cocktails and/or concomitantly with alcoholic beverages.

#### 2.2.2. Global Chemical Reactivity

For the evaluation of chemical reactivity based on the energy levels of the frontier molecular orbitals, *E*_HOMO_ and *E*_LUMO_, several global reactivity descriptors such as, electronegativity (*χ*), hardness (*η*), chemical softness (*S*), chemical potential (*ε*) and electrophilicity index (*ω*), were computed, according to Equations (6)–(10) [47,48,49].

(6) $$\chi =-\frac{E_{\mathrm{LUMO}}+E_{\mathrm{HOMO}}}{2}$$

(7) $$\eta =\frac{E_{\mathrm{LUMO}}-E_{\mathrm{HOMO}}}{2}$$

(8) $$S=\frac{1}{\eta }$$

(9) $$\varepsilon =\frac{E_{\mathrm{LUMO}}+E_{\mathrm{HOMO}}}{2}$$

(10) $$\omega =\frac{\varepsilon^{2}}{2\eta }$$

Additionally, ionization potential (*I*) and electron affinity (*A*) are defined as -*E*_HOMO_ and -*E*_LUMO_, respectively [48,49], where *I* represents the energy needed to remove an electron from a molecule and *A* the energy released when a proton is added into system [48].

The atom power to attract electrons is defined by electronegativity (*χ*), and hardness (*η*) is a measure for both, stability, and reactivity while chemical softness (*S*) represents the inverse of hardness [49]. The electrophilicity index (*ω*) expresses the molecule predisposition to accept electrons [49]. Consequently, nucleophilic agents are characterized by a low electrophilicity index (*ω*), and good electrophilic reagents by a high electrophilicity index. The values of molecular descriptors are listed in Table 4.

Analyzing the values of the molecular descriptors shown in Table 4, it is observed that epirubicin has the highest electronegativity (*χ*) and the highest chemical softness (*S*), respectively and consequently the lowest hardness (*η*), explaining also its interactions with ethyl alcohol. The electrophilicity index (*ω*) shows that paclitaxel represents the best nucleophilic agent compared two epirubicin and gemcitabine, but weaker than limonene.

The drug molecules containing certain atoms with partially negative high charge determines the appearance of a large number of donor–acceptor interactions and, therefore, a better ligand–receptor interaction. Thus, the electric dipole moment (*μ*) is another molecular parameter resulting from quantum chemical calculations, that reflects the partial separation of the electric charge inside the molecule. This descriptor is also a predictor of the chemical reactivity of molecules, indicating the molecular system polarity. As can be seen, the highest value of dipole moment belongs to gemcitabine, which could explain its stronger molecular assembly with limonene compared to those of EPR-LIM and PTX-LIM.

#### 2.2.3. Limonene-Chemotherapeutic Agent Interactions

**Figure 7:**
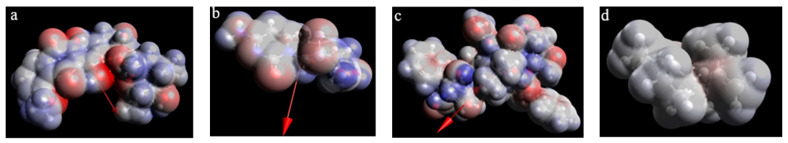
The electrostatic potential of the studied chemotherapeutic agents and limonene: (**a**)—epirubicin; (**b**)—gemcitabine; (**c**)—paclitaxel; (**d**)—limonene.

Based on the molecular property called electrostatic potential, the three-dimensional map of the electronic density was obtained. As shown in Figure 7, values of the electrostatic potential are marked with different colors, indicating the polarity of a certain region on the Van der Waals surface of the molecule. The areas with high potential values will strongly attract polar molecules, and on the contrary, those with low potential values do not have this property, being considered hydrophobic.

The three-dimensional model, indicating the spatial distribution of electrons in chemical molecules, highlights the size and shape of the molecules excluding steric hindrance, thus allowing a better interaction. Additionally, designing of reactive centers on the molecular surface corresponding to the most negative values of the molecular electrostatic potential are marked (Figure 7—blue and red).

The values of limonene-chemotherapeutic agent binding energies were also calculated and listed in Table 5.

The most stable molecular assembly, with free binding energy of −0.37 kcal mol^−1^, results from the limonene interactions with gemcitabine. Epirubicin–limonene molecular complex (free binding energy −0.3 kcal mol^−1^) is less stable than that based on GCT-LIM bonds, and the PTX-LIM assembly does not form spontaneously, the binding free energy reaching a positive value of 1.14 kcal mol^−1^. As it is known, favorable processes have negative values of ΔG, while unfavorable reactions have ΔG positive [50]. This does not exclude PTX-LIM interactions, but without spontaneously organizing into a stable molecular complex.

This was also confirmed by kinetic data showing that the rate constant of GCT decomposition reaction in the essential oil-containing supporting electrolyte (ESO) decreases about of 2.5 times compared to that obtained in the absence of limonene (SE). Thus, a more stable molecular microaggregate was formed than in the case of EPR, when the rate constant of its decomposition reaction in ESO is of 1.3 times lower than in SE. In the case of PTX decomposition reaction, the rate constant has increased about 1.3 times, from 0.0317 uA min^−1^ (in SE) to 0.0412 uA min^−1^ (ESO), proving that a stable molecular assembly of the type PTX-LIM does not form spontaneously, the PTX-LIM interactions are based on labile bonds that appear spontaneously, transiting the formation-breaking cycles, as also reflects the spectra from Figure 3c. On the other hand, in the case of both epirubicin and gemcitabine, the half-life increases approximately 1.4-fold in the presence of limonene, while paclitaxel half-life decreases about of 1.4 times, under the same conditions.

The π-effects are associated with the interactions of molecules with conjugated double bond systems. Additionally, π-alkyl interactions involve the C-H interaction with the π system, this type being successfully studied using experimental techniques and computational methods.

**Figure 8:**
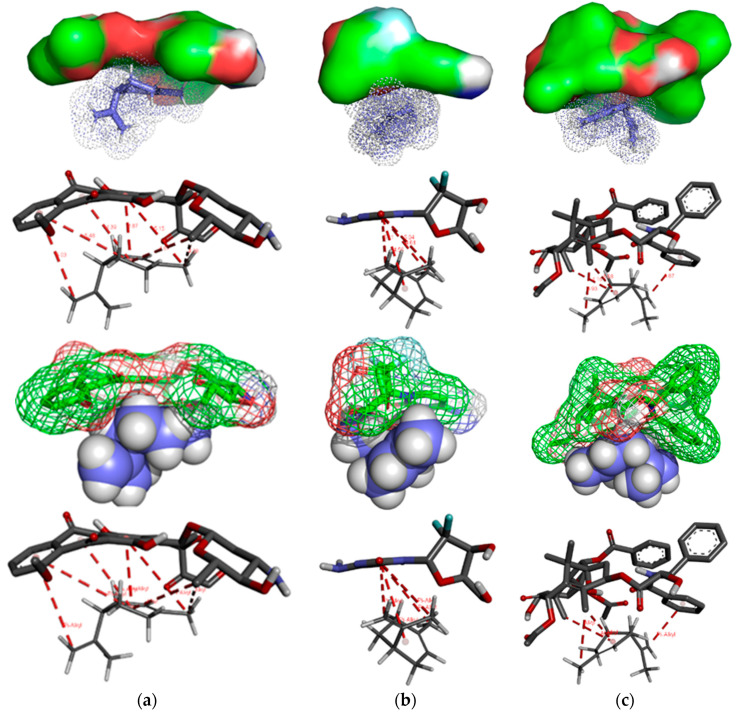
Visualization of the chemotherapeutics-limonene interactions: (**a**) epirubicin–limonene (EPR-LIM); (**b**) gemcitabine–limonene (GCT-LIM); (**c**) paclitaxel–limonene (PTX-LIM).

The alkyl-alkyl interactions represent on the hydrophobic ones, showing the tendency of non-polar molecules to aggregate in aqueous solutions in order to separate from water, leading to the formation of a minimal surface of non-polar molecules exposed to polar water molecules (usually spherical droplets). The hydrophobic effect is not considered a non-covalent interaction, as it is depending on entropy and not a specific interaction between two molecules, usually characterized by entropy-enthalpy couple. Figure 8 shows the interactions between chemotherapeutics and limonene.

Thereby, there are two hydrophobic (alkyl–alkyl) interactions between epirubicin and limonene located at 4.28 Å and 4.87 Å, respectively, and five π-alkyl interactions at 5.15 Å, 3.87 Å, 4.39 Å, 5.48 Å, and 5.03 Å. The connection of gemcitabine with limonene involves four π-alkyl interactions placed at 4.61 Å, 4.56 Å, 5.04 Å, and 5.11 Å. Additionally, three hydrophobic interactions between limonene and paclitaxel at 3.93 Å, 3.98 Å, 4.16 Å, and the one π-alkyl at 3.87 Å are formed. As can be seen in paclitaxel, hydrophobic bonds predominate (three vs. the one π-alkyl), which explains its randomly interactions with limonene and the low possibility of forming a spontaneous and coherently organized molecular complex.

The occurrence of these interactions is not excluded in living organisms, in the case of simultaneous administration of allopathic treatment and that based on orange essential oil, influencing more or less pharmacokinetics and drug dynamics due to the formation of molecular microaggregates, which randomly cleavage.

After Autodock 4.2.6 and Autodock Vina redocking according to the procedure in material and method section, we obtained very low RMSD values (all of them lower that 0.1 Å), suggesting that our preliminary docking methodology is robust.

The involvement of molecular descriptors on conducting QSAR/QSPR studies allowed deepening the molecular design and implicitly the prediction and description of some properties of epirubicin, gemcitabine and paclitaxel as well as their interaction with limonene. Moreover, the quantum chemical calculations completed information difficult to estimate only from laboratory experiments, mainly highlighting occurrence and type of chemotherapeutic agent–limonene interactions and framing in molecular assemblies with assumed stability depending on the binding energy values computed.

### 2.3. Decomposition Mechanism of Drugs

Based on the information obtained from the theoretical study and according to the data reported in the literature, a decomposition mechanism of the studied compounds was proposed. The decomposition/metabolism processes are sometimes subjected to an unexpected evolution caused by some fortuitous phenomena influencing the appearance of chemical species with a short lifetime, such as free radicals, which by combination or disproportionation can lead to wide range of chemical compounds. The reaction mechanisms proposed for the decomposition of chemotherapeutics are theoretical and take into account possible electrode processes, through which certain unstable and non-insulable intermediates are formed, as further is shown, which by successive stages turn into certain final reaction products that are reported in the literature. Additionally, a certain proportion of the drug can degrade completely to carbon dioxide and water.

#### 2.3.1. Epirubicin Decomposition Mechanism

**Scheme 1:**
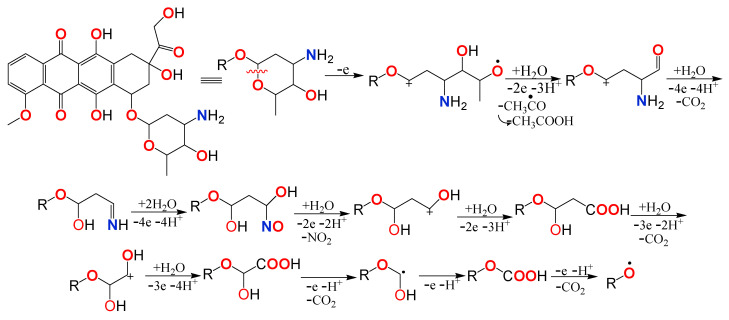
The formation of the intermediate radical of tetracene structure.

Epirubicin has the lowest dipole moment value (1777 D) indicating a high degree of molecular symmetry as well as a low polarity. Additionally, from studied drugs, epirubicin presents the lowest value of the energy gap of frontier orbitals. Thus, EPR molecule has a relative stability of the tetracene structure due to the extended conjugate system. Under these conditions, the first oxidation stage takes place at the oxane ring level. The electrochemical degradation of this O-heterocycle by successive stages of charge transfer to the platinum electrode surface leads to an intermediate, as tetracene structure (Scheme 1), that was identified as a product of epirubicin metabolic degradation [51,52,53,54].

**Scheme 2:**
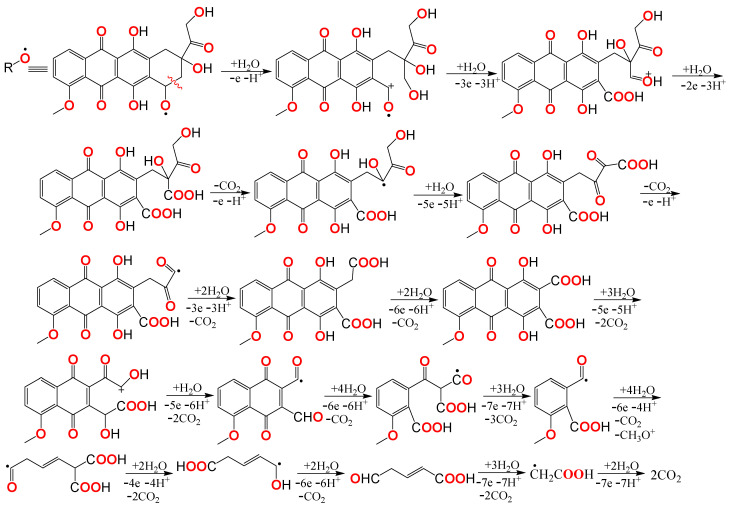
Final degradation of tetracene structure.

The electrochemical degradation of the tetracene structure is a complex process, the mechanism intermediate stages depend on the current density and the rate constants of the protonation/deprotonation reactions. Thus, for the final electrochemical degradation, the successive stages as shown Scheme 2, are proposed.

#### 2.3.2. Gemcitabine Decomposition Mechanism

**Scheme 3:**
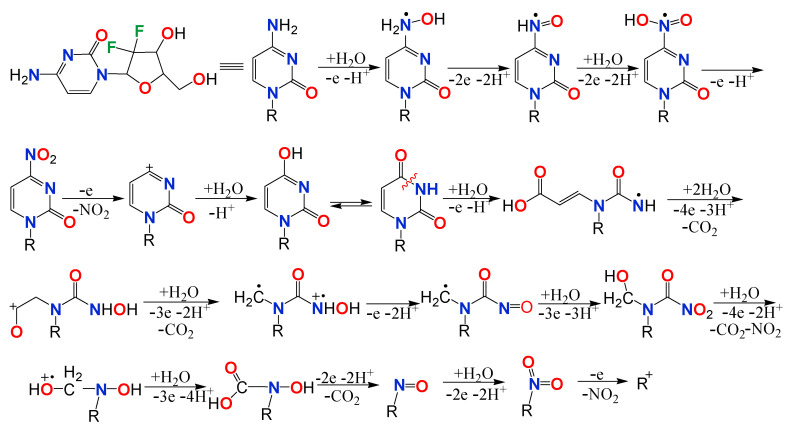
The formation of difluoro deoxy-uridine.

Gemcitabine molecular structure consists of two cycles namely pyrimidine ring and oxolane ring. The presence of π-π and n-π conjugations leads to an extended conjugate system throughout the pyrimidine cycle. Two geminal fluorine atoms are linked from oxolane ring, inducing a great molecule polarity, thus explaining the dipole moment high value of 6598 D. The amino group from the side chain, with a high susceptibility to oxidation, participates to the charge transfer processes, forming the corresponding nitro-derivative. Elevated reactivity of the –NH_2_ group connected to the pyrimidine ring, was also identified by the studies of GCT metabolic degradation products [55,56,57,58]. Oxidative removal of the nitro group leads to the formation of difluoro deoxy-uridine (Scheme 3).

**Scheme 4:**
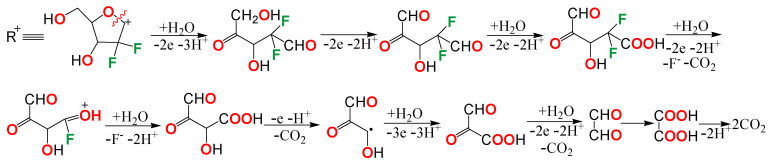
Gemcitabine final degradation.

The conjugations n-π and π-π are also evident in the structure of intermediates. GCT electrochemical degradation by intermediates exhibiting electronic conjugations can explain the appearance of the isosbestic point at 248 nm. The degradation of the intermediates, over time, with the oxidative cleavage of the two cycles (Scheme 4), explains the decrease of GCT absorbance values and the formation of degradation products identified in the UV absorption zone, at small wavelengths of 225–227 nm.

#### 2.3.3. Paclitaxel Decomposition Mechanism

**Scheme 5:**
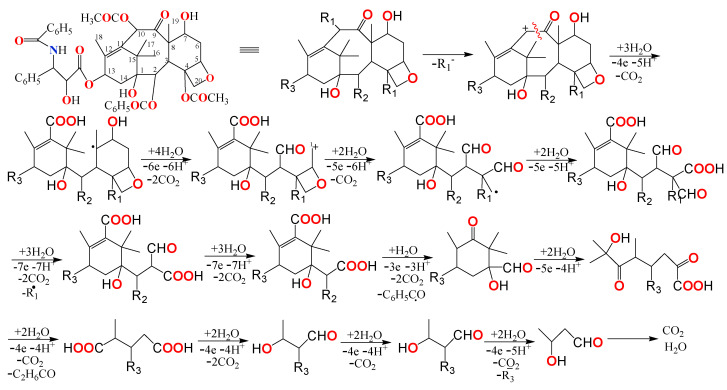
Paclitaxel degradation mechanism.

According to the optimization model of drug molecular structure and frontier orbitals designing, PTX structure displays a high charge density at the level of the atoms from positions from 9 to 12, thus participating to oxidation processes by cleavage of the cycle consisting of eight carbon atoms [59,60,61,62]. Additionally, paclitaxel has in its structure several ester type functional groups, which under the action of electric current can participate in electrode processes by cleaving the C-O bond and releasing carboxyl group as shown Scheme 5, where R_1_ = acetate, R_2_ = benzoate and R_3_ = phenylpropanoate.

Substituents R_1_ are subsequently oxidized to acetate, which by decarboxylation degrades to carbon dioxide and water. Substituents R_2_ and R_3_ contain benzene nuclei, which can participate in electrochemical mineralization processes at the platinum electrode surface.

## 3. Materials and Methods

### 3.1. Materials

The interactions between the drugs listed above and limonene were studied in 5 × 10^−2^ mol L^−1^ hydrochloric acid solution, containing (in volumes) 40% ethyl alcohol and 60% water to ensure a good solubility of limonene in the binary system water/alcohol.

Orange essential oil purchased from Aromaterapia Company, Romania, according to brand analysis report, has a following chemical composition: α-pinene = 0.57%; sabinene = 0.25%; β-myrcene = 2.03%; limonene = 95.86%; β-phellandrene = 0.25%; c8 aldehyde = 0.32%; c9 aldehyde = 0.19%; linalool = 0.36%; and geranial and neral 0.12%.

Injectable solutions of cytostatics stored in specific vials were used, these being marketed by certain companies, as follows: (i) 50 mg epirubicin (EPR)/25 mL solution containing natrium chloride and hydrochloric acid, as excipients (Teva); (ii) 200 mg gemcitabine (GCT)/5 mL solution containing natrium hydroxide and hydrochloric acid, as excipients (Actavis); and (iii) 150 mg paclitaxel (PTX)/25 mL solution containing citric acid, ethyl alcohol, and macrogolglycerol ricinoleate (Actavis).

To obtain a coherent spectrophotometric response, appropriate concentrations of limonene and chemotherapeutics were used for the preparation of reaction media. Their complexity requires additional specifications as well as specific designations, as follows: (1) blank electrolyte: 5 × 10^−2^ mol L^−1^ hydrochloric acid solution; (2) 5 × 10^−2^ mol L^−1^ hydrochloric acid solution containing, in turn, each studied cytostatic (CYT_HCl) noted as follows, EPR_HCl, GCT_HCl and PTX_HCl, respectively; (3) support electrolyte (SE) consisting of 5 × 10^−2^ mol L^−1^ hydrochloric acid in ethyl alcohol/water solution, in the ratio of 2/3 volume; (4) SE containing the studied cytostatics (CYT_SE), respectively (EPR_SE), (GCT_SE) and paclitaxel (PTX_SE); (5) SE with the addition of 0.01 mL of orange essential oil in 1000 mL SE (ESO), obtaining a limonene concentration of 7 × 10^−5^ mol L^−1^; and (6) ESO with the addition of each cytostatic, separately (CYT_ESO), respectively EPR_ESO, GCT_ESO and PTX_ESO.

The same concentration of the drugs was used, in all studied environments namely: epirubicin—2.57 × 10^−4^ mol L^−1^, gemcitabine—1.52 × 10^−4^ mol L^−1^, and paclitaxel—1.12 × 10^−5^ mol L^−1^. The bi-distilled water was used for the preparation of the solutions, ethyl alcohol obtained from Tunig distributor, Romania, and hydrochloric acid with analytical purity grades being purchased from SC Silal Trading, Romania.

### 3.2. Preparation of Work Environments

The working protocol involved several stages.

(1) preparation of stock solutions (1000 mL), as follows:

EPR_HCl: 100 mL EPR (2 mg mL^−1^), 50 mL of 1 mol L^−1^ HCl solution, and water up to 1000 mL, thus obtaining the EPR concentration of 0.2 g L^−1^ in 5 × 10^−2^ mol L^−1^ HCl solution;

GCT_HCl: 1 mL GCT (40 mg mL^−1^), 50 mL of 1 mol L^−1^ HCl solution, and water in balance, resulting 0.04 g L^−1^ GCT in 5 × 10^−2^ mol L^−1^ HCl solution;

PTX_HCl: 2 mL GCT (6 mg mL^−1^), 50 mL of 1 mol L^−1^ HCl solution, and water in balance, the composition consisting of 0.012 g L^−1^ GCT in 5 × 10^−2^ mol L^−1^ HCl solution.

**Figure 9:**
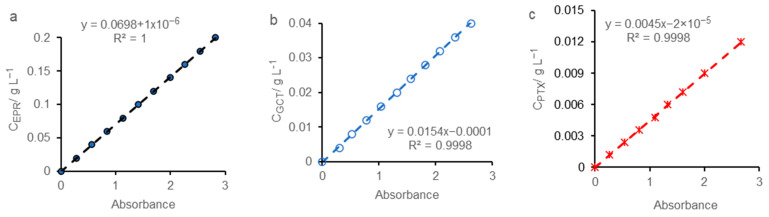
Calibration curve recorded for epirubicin (**a**), gemcitabine (**b**), and paclitaxel (**c**) in 5 × 10^−2^ mol L^−1^ HCl solution.

(2) By successive dilutions with 5 × 10^−2^ mol L^−1^ HCl solution, the media corresponding to each chemotherapeutic were generated and submitted for spectrophotometric analysis. Thus, the calibration curves shown in Figure 9 were drawn.

(3) Thus, using the equations inserted in Figure 9a–c, the initial concentrations of the drugs were computed considering the spectral accuracy of the maximum absorbance. Thus, using the equations inserted in Figure 9a–c, the initial concentrations of the drugs were computed considering the spectral accuracy of the maximum absorbance. Additionally, the optimal volume of cytostatic solutions, which was further used for the preparation of all studied media was established. (Table 6).

(4) The essential orange oil was 100 times diluted with ethyl alcohol, obtaining a solution of concentration of 0.9586% in limonene (DESSO), from which 1 mL was used, for the preparation of 1000 mL from each working environment, respectively EPR_ESO, GCT_ESO, and PTX_ESO, the final limonene concentration reaching the value of 0.9586 × 10^−2^ g L^−1^ meaning 7 × 10^−5^ mol L^−1^. Basically, the orange oil was diluted by 10,000 times.

(5) Based on the above, the composition of the reaction media used in this study are systematized in Table 7.

It is known that the concentration (C) is directly proportional to the absorbance (A), that means whatever a concentration (Ci) respects the relation, Ci = ζAi, where ζ is a proportionality factor representing the straight-line slope resulted from the graph C = f(A), as shown Figure 9, in respect with the value of coefficient R^2^, which must be close to the unity. Therefore, the following values for ζ is deduced from calibration curves, namely 0.0698 for EPR, 0.0154 for GCT and 0.0045 for PTX, respectively. Consequently, the absorbance is used as a parameter for description, analysis, and kinetic calculation.

### 3.3. Investigation Methods

#### 3.3.1. UV-Vis Spectrophotometry

UV-Vis spectra were recorded using a Varian Cary 40 spectrophotometer, with CaryWin software, in the wavelength (λ) range between 800 nm and 200 nm. During electrolysis the spectra were recorded at certain intervals imposed by the degradation rate of the studied compounds, as follows: (i) EPR_HCl every two minutes for 30 min, EPR_SE and EPR_ESO every four minutes for one hour; (ii) GCT_HCl and GCT_SE minute by minute for 10 min and 16 min, respectively, and GCT_ESO every two minutes for 16 min; and (iii) PTX_HCl every two minutes, time of 20 min and for PTX_SE solution, every four minutes, time of 40 min; for the response accuracy, PTX_ESO spectra were recorded at the following time intervals: initially, 8, 12, 16, 18, 22, 26, and 30 min. The analysis report accurately signaled the absorbance values at the wavelengths corresponding to each analyzed compound.

#### 3.3.2. Electrochemical Measurements

The experiments were performed in a standard electrochemical cell with two identical platinum electrodes having an active area of 1 cm^2^. The electrolysis was performed using a potentiostat/galvanostat VoltaLab 40 with VoltaMaster4 software, maintaining the constant current density at 50 mA cm^−2^, for a determined time by the electrochemical degradation reaction of the studied compounds.

In our previous studies [43,44,63,64,65], the same methodology based on electrochemical measurements assisted by UV-Vis spectrophotometry was used to investigate the stability and interactions, respectively, of some pharmaceutical compounds in multicomponent systems such as, valproic acid [63], ceftriaxone [43], benzocaine [64], vitamin A [65], and vitamin C [44].

### 3.4. Theoretical Study

#### 3.4.1. Molecular Modelling

All structures were optimized using Gaussian 09 software (Gauss View 16 interface) by the DFT/B3LYP/6-311G method and subsequent, for each compound the molecular descriptors were computed.

#### 3.4.2. Docking Protocol

Autodock 4.2.6 is an automatic procedure to predict the interaction between two or more molecules. It aims to obtain the global minimum energy of interaction between two compounds, exploring all the degrees of system freedom. Autodock 4.2.6 combines two methods to achieve this objective namely, fast energy scanning and efficient space evaluation. Subsequent, the conformations are assessed using semi-empirical force fields. The force field includes six pairs of assessments and an estimate of conformational entropy after docking, Relationship 11.

⊗G = [V_(boundL-L)_ − V(_unboundL-L_)] + [V(_boundT-T_) − V(_unboundT-T_)] + [V(_boundT-L_) − (V_unboundT-L_) + ⨂_Sconf_] (11)

where L refers to the ligand and T to the target structure (another molecule that interacts with the ligand), in a molecular docking calculation.

Each pair of energy terms includes the evaluation of dispersion/repulsion, hydrogen bonds, electrostatic interaction, and dissolution. The free binding energy (ΔG) was calculated with Equation (12).

ΔG = ΔH − TΔS(12)

where ΔH represents the enthalpy and TΔS the entropic contribution (only the negative value of ΔG is energetically favorable and the process is spontaneous) [43].

All the hydrogen atoms to the substances were added and Gasteiger charge was selected.

In the grid stage, the grid box of 40 × 40 × 40 has been set at a distance of 0.375 Å from the limonene center. In the docking Lamarckian Genetic Algorithm (LGA) was chosen, with a population size of 150, a maximum of 2.5 × 106 energy evaluations, and a number of 30 runs. The images of the limonene–chemotherapeutic complexes were viewed using the PyMol software (PyMOL Molecular Graphics System, Version 2.0 Schrödinger, LLC, New York, NY, USA) and Discovey Studio ((BIOVIA, Dassault Systèmes, BIOVIA Workbook, Release 2017; BIOVIA Pipeline Pilot, Release 2017, San Diego: Dassault Systèmes, 2019, San Diego, CA, USA)).

We evaluated the performance of the molecular docking tool by performing the calculations in triplicate and then expressing the results and the root-mean-square deviation (RMSD) as averages. The redocking involves the overlapping of the ligands for calculating the RMSD with the Discovery Studio software. We also performed a comparative RMSD analysis between Autodock 4.2.6 and AutoDock Vina to assess the docking method’s repeatability and reproducibility.

## 4. Conclusions

UV-Vis spectroscopy revealed that a strong interaction between epirubicin and ethyl alcohol occurs, pointing out that the ethanol interaction with both gemcitabine and paclitaxel is less perceivable. The orange essential oil presence in alcoholic hydrochloric acid solution causes spectral changes of the three studied drugs, showing that certain types of interactions between chemotherapeutics and limonene have occurred.

Orange essential oil influences both the decomposition rate and the half-life of the studied compounds, which involves certain interactions between cancer drug and the essential oil components, especially with the majority compound namely limonene.

Additionally, this acts as an inhibitor of both epirubicin and gemcitabine electrochemical decomposition reaction and potentiates the paclitaxel decomposition reaction in a supporting electrolyte.

Retarding effect on the decomposition reaction of epirubicin and gemcitabine is due to some interactions that favored the spontaneous formation of drugs–limonene molecular micro-assemblies. The essential orange oil quickly affected paclitaxel chemical stability still from the first contact of the two compounds. The interactions between limonene and paclitaxel are based on random bonds that do not have the strength to organize the two compounds into a stable complex.

Kinetic data confirmed the spectrophotometric analysis, showing that the limonene addition in SE versus its absence, led to the rate constants decrease and the half-lives increase, in the case of epirubicin and gemcitabine, and it induced an increase in the rate constant and a decrease in half-life for paclitaxel.

The theoretical study showed that certain bonds were formed between the studied drugs and limonene namely, two hydrophobic and five π-alkyl EPR-LIM interactions, four π-alkyl GCT-LIM interactions and finally, three hydrophobic and the one π-alkyl PTX-LIM interactions.

The molecular docking technique allowed the visualization of the interactions between drugs and limonene, as well as the determination of the binding free energy (ΔG). Consequently, the most stable molecular complex was formed between gemcitabine and limonene having a free binding energy value of −0.37 kcal mol^−1^, followed by that formed between epirubicin and limonene (ΔG = −0.3 kcal mol^−1^). The spontaneous formation of the PTX-LIM molecular complex is an unfavorable process, ΔG reaching a positive value of 1.14 kcal mol^−1^.

Although in the human body such drastically current-potential conditions are not reached, the simultaneous administration of alternative treatments with allopathic ones should respect a consistent medical protocol, in order to avoid the implications on the chemotherapeutic drug biological response. Additionally, the instantaneous interactions that can occur between the chemical species, in certain multicomponent systems, can lead to the appearance of other toxic compounds affecting human health.

Our study can be indicative, revealing a trend regarding the appearance of interactions between the studied drugs and the essential oil components, especially limonene, which were predicted using the automatic procedure Autodock 4.2.6. These have led to changes in the chemical kinetics of their decomposition reaction and could, therefore, influence to a greater or lesser extent, the metabolic breakdown of drugs.

## Figures and Tables

**Table 1:** Molecular formulas and UV-Vis spectra of chemotherapeutics recorded in the supporting electrolyte without and with orange essential oil.

Molecular Structures of Drugs	UV-Vis Spectra of Compounds in Studied Media	λ_max_/nm
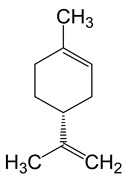 Limonene (LIM); C_10_H_16_; M = 136.23 g mol^−1^	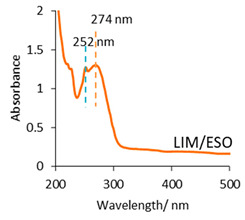	LIM 274 nm 252 nm
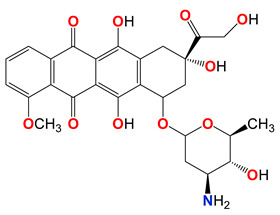 Epirubicin (EPR); C_27_H_29_NO_11_ × HCl; M = 543.174 g mol^−1^	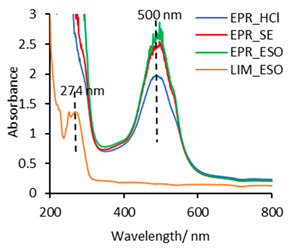	EPR 500 nm
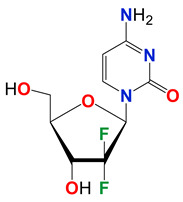 Gemcitabine (GCT); C_9_H_11_F_2_N_3_O_4_; M = 263.2 g mol^−1^	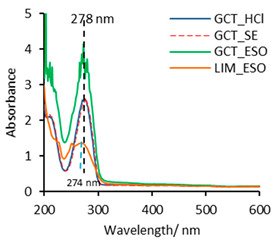	GCT 278 nm
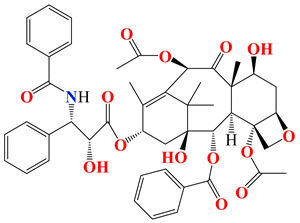 Paclitaxel (PTX); C_47_H_51_NO_14_; M = 853.331 g mol^−1^	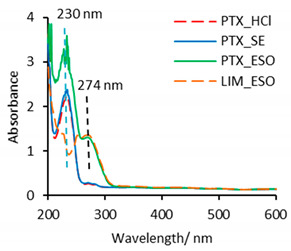	PTX 230 nm

**Table 2:** The experimental and computed absorbance values from applied kinetic models according to the displayed coefficient R^2.^

CYT/Environment	First-Order Reaction Kinetics			Zero-Order Reaction Kinetics		
	A_o_/Experimental	A_o_/Computed from A_o_= Ae^−kt^	R^2^	A_o_/Experimental	A_o_/Computed from A_o_ = A − kt	R^2^
EPR_HCl	2.01	2.07	0.996	-	-	-
EPR_SE	2.54	2.38	0.988	-	-	-
EPR_ESO	2.86	2.35	0.967	-	-	-
GCT_HCl	-	-	-	2.62	2.64	0.997
GCT_SE	-	-	-	2.6	2.55	0.994
GCT_ESO	4.12	4.16	0.994	4.16	3.93	0.960
PTX_HCl	-	-	-	2.21	2.16	0.998
PTX_SE	-	-	-	2.34	2.28	0.994
PTX_ESO	-	-	-	2.11	2.12	0.995

**Table 3:** Kinetic parameters computed for the electrodecomposition reaction of EPR, GCT and PTX in studied environments.

CYT/Environment	First Order Reaction Kinetics			Zero Order Reaction Kinetics	
	ln(A_o_/A) = kt	A = A_o_e^−kt^	t_1/2_ = (ln2)/k	A = A_o_ − kt	t_1/2_ = A_o_/2k
	k (min^−1^)		t_1/2_ (min)	k (uA min^−1^)	t_1/2_ (min)
EPR_HCl	0.062	0.0621	11.2	-	-
EPR_SE	0.015	0.0152	46.2	-	-
EPR_ESO	0.011	0.0109	63.0	-	-
GCT_HCl	-	-	-	0.3114	4.2
GCT_SE	-	-	-	0.2065	6.2
GCT_ESO	0.081	0.081	8.6	-	-
PTX_HCl	-	-	-	0.069	15.7
PTX_SE	-	-	-	0.0317	35.9
PTX_ESO	-	-	-	0.0412	25.8

**Table 4:** Quantum chemical parameters calculated for studied cytostatics and limonene.

Descriptors	Chemical Compound			
	EPR	GCT	PTX	LIM
E_HOMO_	−9.049	−9.673	−8.863	−9.181
E_LUMO_	−1.657	−0.559	−0.387	1.345
I/eV	9.049	9.673	8.863	9.181
A/eV	1.657	0.559	0.387	−1.345
ΔE/eV	7.392	9.114	8.476	10.256
χ/eV	5.353	5.116	4.625	3.918
η/eV	3.696	4.557	4.238	5.263
S/eV^−1^	0.271	0.219	0.236	0.19
ε/eV	−5.353	−5.116	−4.625	−3.918
ω/eV	3.87	2.87	2.52	1.45
Dipole moment/D	1.77	6.598	3.67	0.904

**Table 5:** Values of chemotherapeutics-limonene binding energies.

Binding Energy (Kcal mol^−1^)	Chemotherapeutic Agent-Limonene		
	EPR-LIM	GCT-LIM	PTX-LIM
Final intermolecular energy	−3.58	−1.86	−3.93
Final total internal energy	−4.59	−1.99	−10.06
Torsion free energy	+3.28	+1.49	+5.07
Free binding energy of chemotherapeutic agent-limonene (ΔG)	−0.30	−0.37	+1.14

**Table 6:** Spectral parameters and quantitative values calculated for each drug.

Drug	λ_max_/nm	A	C/g L^−1^	C/mol L^−1^	Volume of Drug Solution
Epirubicin	500	2.01	0.14	2.57 × 10^−4^	70
Gemcitabine	278	2.62	0.04	1.52 × 10^−4^	1
Paclitaxel	230	2.21	0.01	1.17 × 10^−5^	1.7

**Table 7:** The composition of studied environments.

Solution	Volume from Basic Drug Solution/mL	5 × 10^−2^ mol L^−1^ HCl Solution/mL	DESSO/mL	Water/mL	Ethanol/mL
EPR_HCl	70	50	-	880	-
EPR_SE	70	50	-	480	400
EPR_ESO	70	50	1	481	399
GCT_HCl	1	50	-	880	-
GCT_SE	1	50	-	480	400
GCT_ESO	1	50	1	481	399
PTX_HCl	1.7	50	-	880	-
PTX_SE	1.7	50	-	480	400
PTX_ESO	1.7	50	1	481	399

## Data Availability

Data is contained within the article.
